# Supplementary material for: Sensory TRP channels contribute differentially to skin inflammation and persistent itch
Source: Nat Commun. 2017 Oct 30;8:980. doi: 10.1038/s41467-017-01056-8 (PMC5661746; doi:10.1038/s41467-017-01056-8)
Supplement: Supplementary file 1 — Supplementary information [file 41467_2017_1056_MOESM1_ESM.pdf]

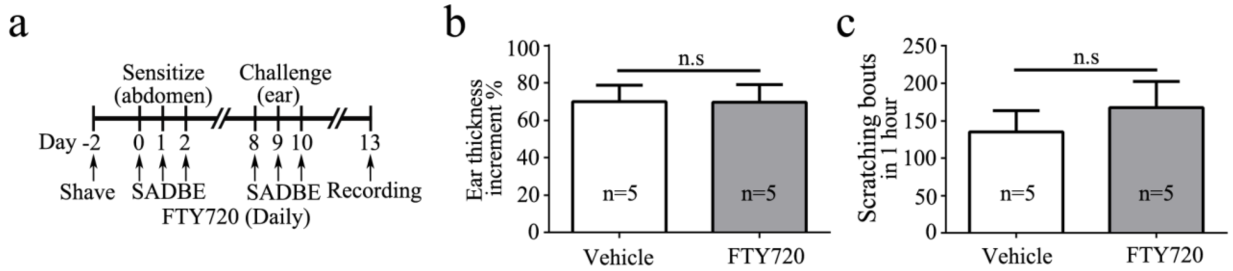

Supplementary Figure 1. Pharmacological inhibition of T cell egression with FTY720 affects neither skin inflammation nor spontaneous scratching. (**a**) Shematic protocol of FTY720 treatment in the induction of SADBE-induced CHS. (**b-c**) Ear thickness increment (**b**) and spontaneous scratching (**c**) in *wt* mice when compared with vehicle-treated animals. Data are presented as mean ± SEM. n.s, not significant, Student's *t*-test.

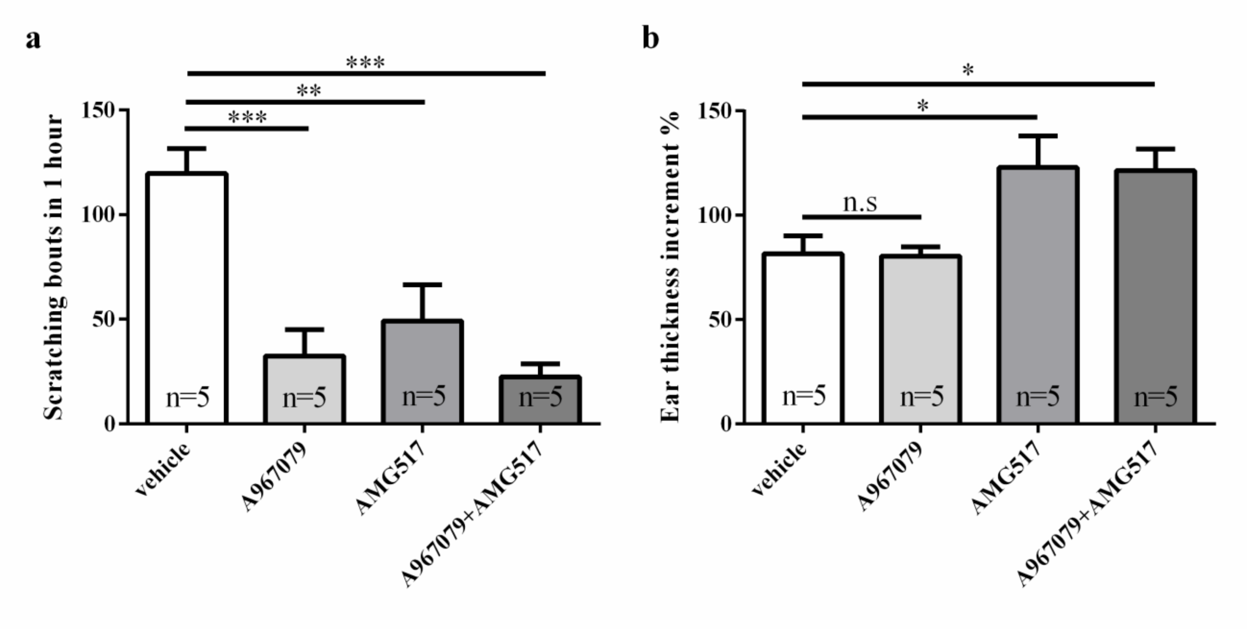

Supplementary Figure 2. Differential effects of pharmacological inhibition of TRPA1 and/or TRPV1 on SADBE-induced skin inflammation and spontaneous scratching. (**a-b**) SADBE-elicited spontaneous scratching (**a**) and skin inflammation (**b**) in *wt* mice treated with selective TRPA1 and/or TRPV1 antagonists. Mice were given 200 µl of 10 mg/kg A967079 (a potent and selective TRPA1 antagonist) and/or 30 mg/kg AMG517 (a potent and selective TRPV1 antagonist) by oral gavage daily starting 3 days before the first SADBE challenge. Itch behavior and ear edema were measured 3 days after the last SADBE challenge. Data are presented as mean ± SEM. n.s, not significant, * $p<0.05$, ** $p<0.01$, *** $p<0.001$, ANOVA.

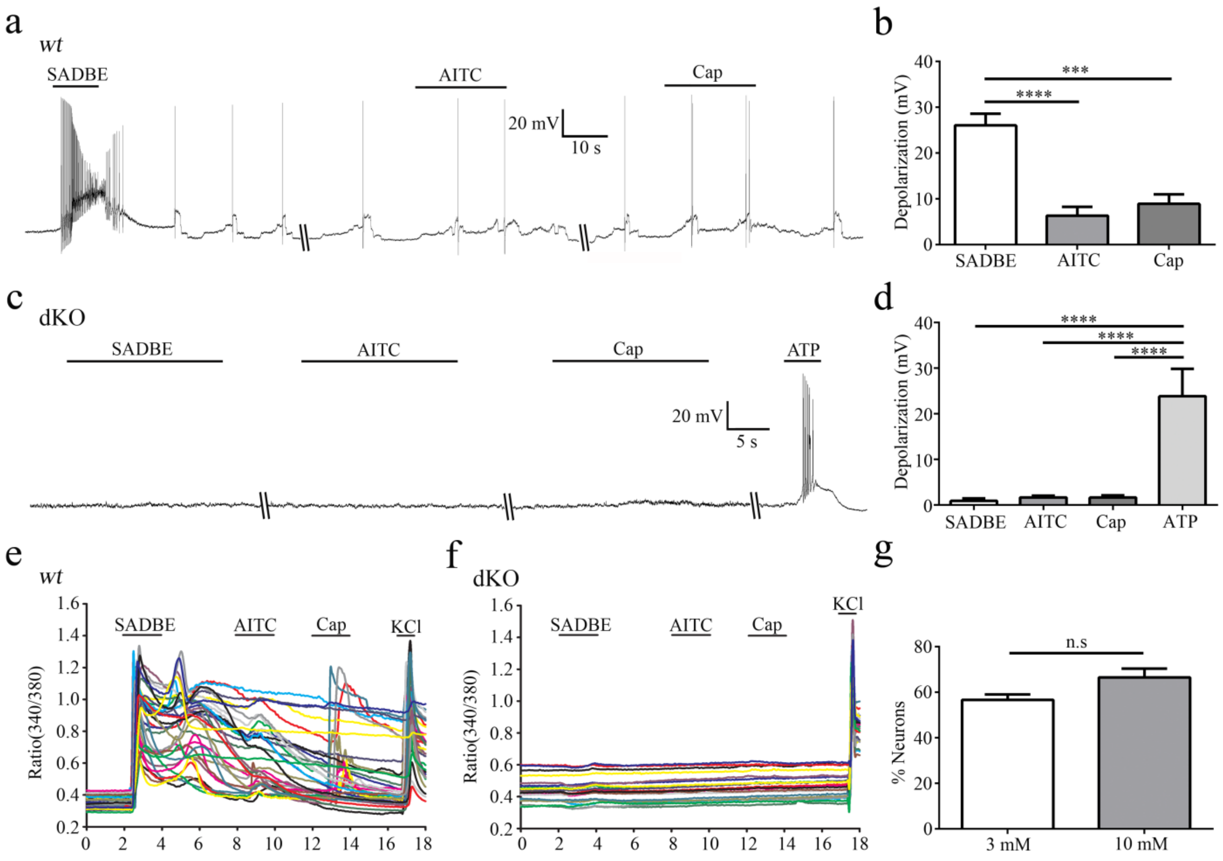

Supplementary Figure 3. SADBE at 10 mM does not activate DRG neurons isolated from the *Trpa1*$^{-/-}$/*Trpv1*$^{-/-}$ dKO mice. (**a**) SADBE-induced depolarization of membrane potential and action potential firing in DRG neurons isolated from *wt* mice; (**b**) Quantification of membrane depolarization induced by SADBE, AITC and Cap. Data are presented as mean ± SEM. n=5, *** *p*<0.001, **** *p*<0.0001, ANOVA; please note that AITC- and Cap-activated responses were markedly reduced when applied after SADBE. (**c**) SADBE did not induce depolarization of membrane potential and action potential firing in DRG neurons isolated from the *Trpa1*$^{-/-}$/*Trpv1*$^{-/-}$ dKO mice; ATP was used as a positive control; (**d**) Quantification of depolarization of membrane potentials induced by SADBE, AITC, Cap and ATP. Data are presented as mean ± SEM. n=5, **** *p*<0.0001, ANOVA; (**e-f**) SADBE-induced calcium influx in *wt* (n=5 coverslips, 689 neurons) and *Trpa1*$^{-/-}$/*Trpv1*$^{-/-}$ dKO (n=5 coverslips, 752 neurons) DRG neurons; (**g**) Percentages of *wt* DRG neurons responded to 3 mM and 10 mM SADBE. Data are presented as mean ± SEM. n.s, not significant. Student's *t*-test.

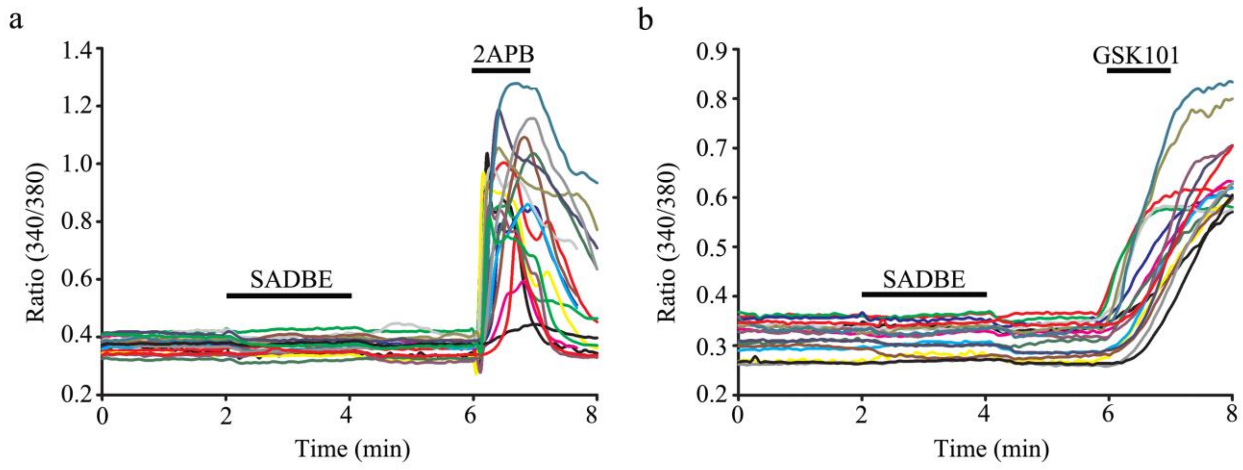

Supplementary Figure 4. SADBE does not activate HEK293 cells transfected with mouse TRPV3 or rat TRPV4 construct. (**a**) 3 mM SADBE did not induce calcium influx in HEK293 cells transfected with mouse TRPV3; the TRPV4 activator 300 µM 2-Aminoethoxydiphenylborane (2APB) was used as a positive control; (**b**) 3 mM SADBE did not induce calcium influx in HEK293 cells transfected with rat TRPV4; the TRPV4 activator GSK1016790A (GSK101, 0.3 µM) was used as a positive control.

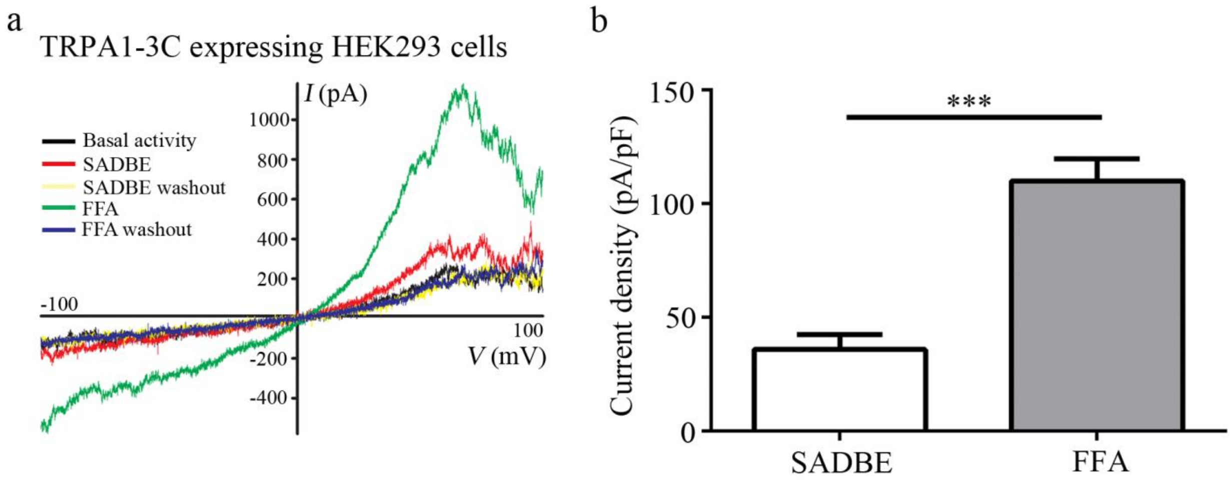

Supplementary Figure 5. SADBE-activated whole-cell membrane currents are severely attenuated in TRPA1 cysteine mutants expressed in HEK293 cells. (**a**) Representative I-V curves of TRPA1-3C currents in response to 3 mM SADBE and 100 µM FFA; (**b**) Quantification of SADBE- and FFA-induced TRPA1-3C currents measured at +60 mV. FFA is a non-electrophilic TRPA1 activator. Data are presented as mean ± SEM. n=6. *** $p<0.001$, Student's *t*-test.

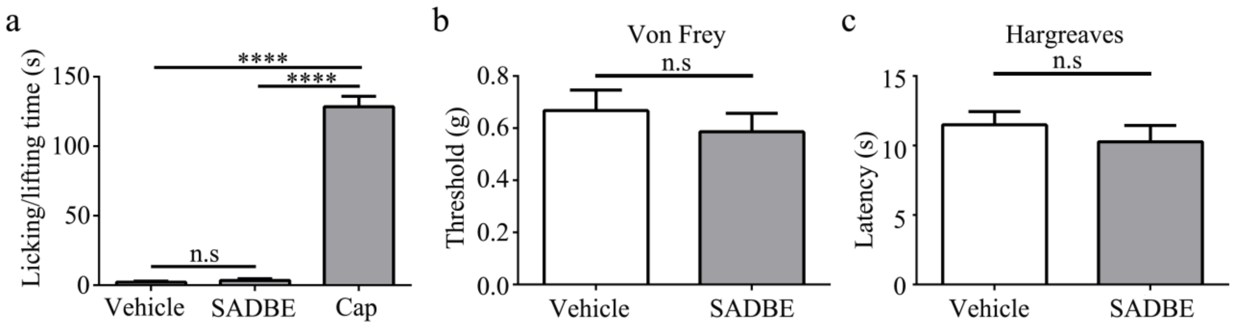

Supplementary Figure 6. Intraplantar injections of SADBE does not elicit a pain-like behaviors in mice. (**a**) Paw licking/lifting nocifensive response after intradermal injection of vehicle, SADBE (30mM) or capsaicin (Cap, 0.5 µg) into a hindpaw of *wt* mice. Data are presented as mean ± SEM. n=5 per group. Asterisks indicate statistical significance. **** $p<0.0001$, ANOVA. n.s, not significant. (**b**-**c**) Paw withdrawal threshold in response to mechanical stimuli (**b**) and thermal stimuli (**c**) after intraplantar injections of 10 µl vehicle or 30 mM SADBE in *wt* mice. Data are presented as mean ± SEM. n=5 per group. n.s, not significant, Student's *t*-test;

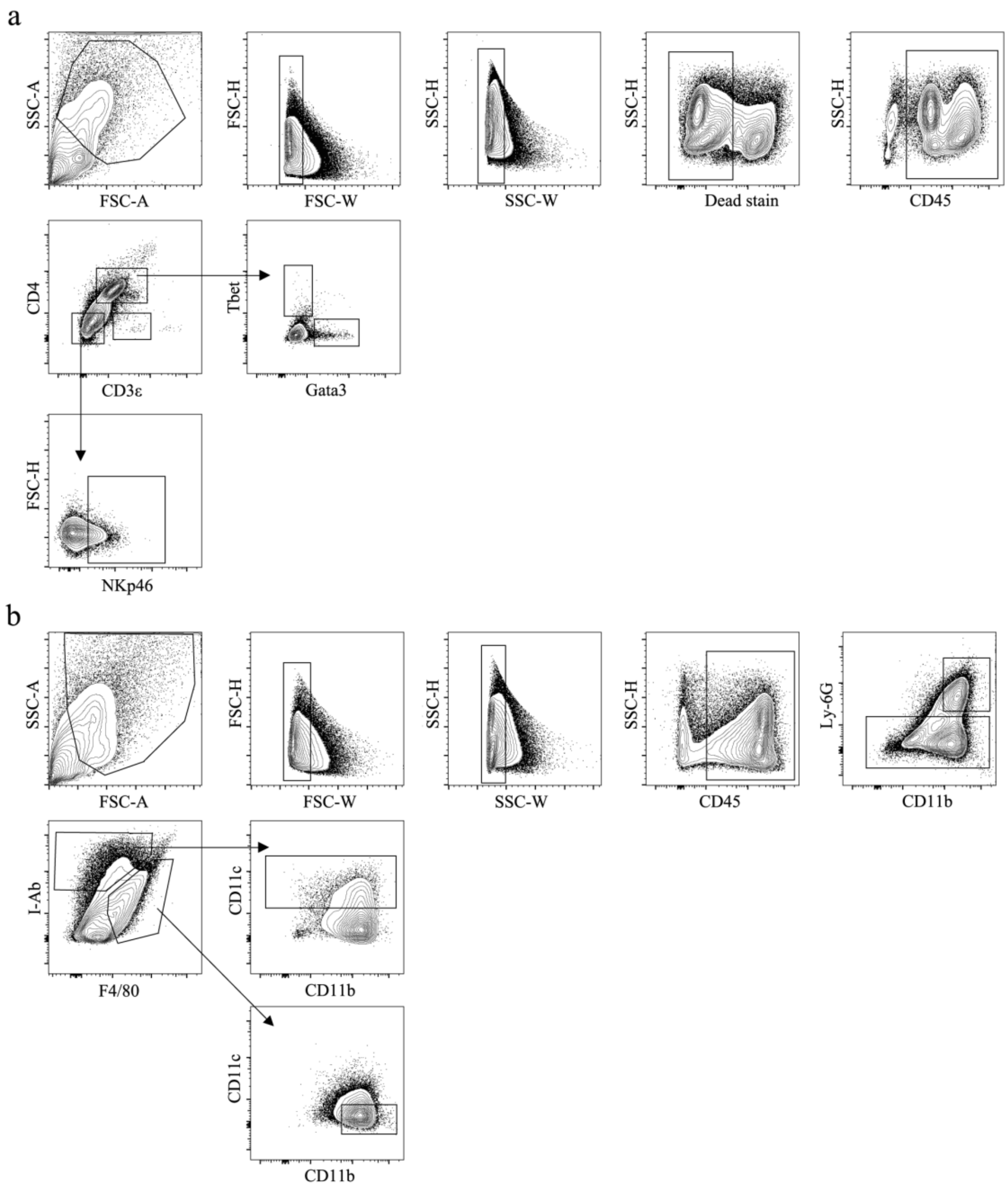

Supplementary Figure 7. Gating strategy for flow cytometry using single-cell suspensions from mouse ear skin preparations. (a) Cells were gated on size and granularity, doublet events and dead cells removed, and then CD45^{+} cells were selected. CD11b^{+} Ly-6G^{+} cells were used to define neutrophils. Ly-6G^{-} cells were further divided into F4/80^{hi} I-Ab^{low} macrophages and F4/80$^{low/-}$ I-A$^{b\text{-}hi}$ cells dendritic cells. Macrophages and dendritic cells were further classified as CD11c^{-} and CD11c^{+}, respectively; (b) Helper T cells were defined as CD3ε^{+} CD4^{+} lymphocytes expressing either Gata3 (Th2) or Tbet (Th1) transcription factors by intracellular staining. NK cells were defined as CD3e^{-} CD4^{-} lymphocytes that were NKp46^{+}.

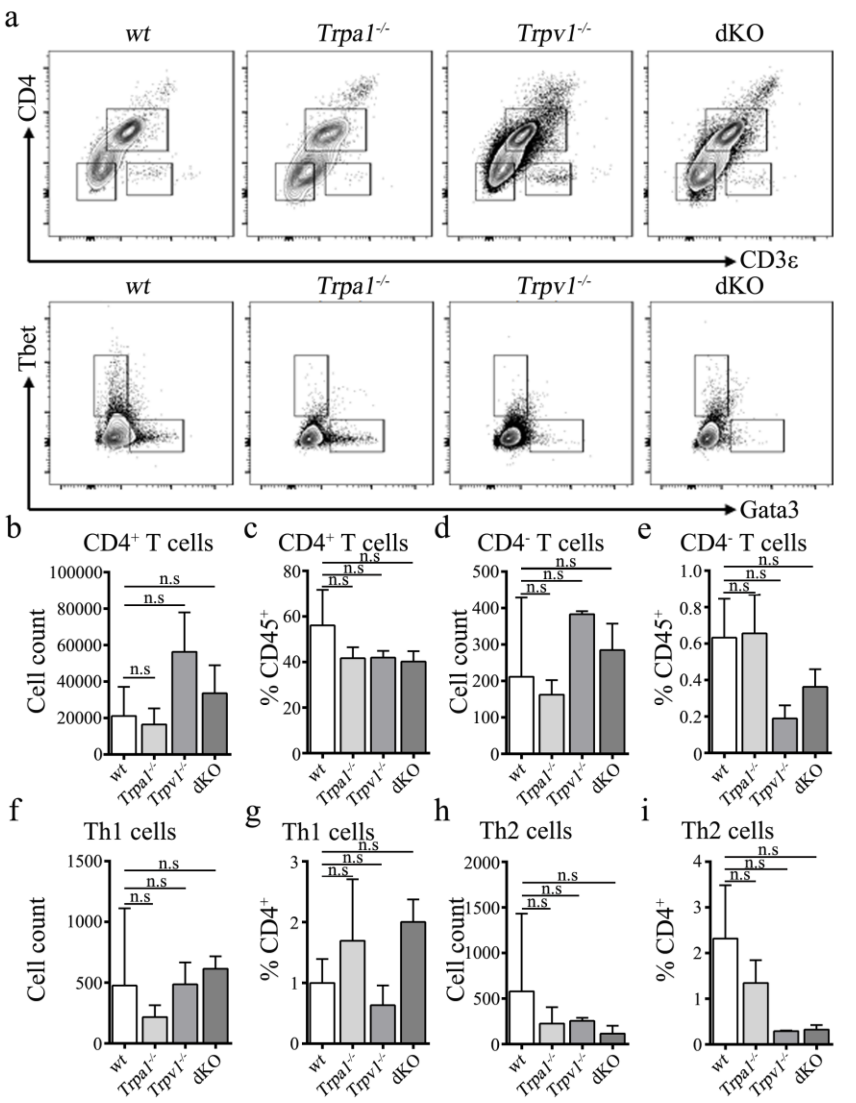

Supplementary Figure 8. Quantification of T cell populations in SADBE-treated ear preparations from *wt*, *Trpa1*$^{-/-}$, *Trpv1*$^{-/-}$ and *Trpa1*$^{-/-}$/*Trpv1*$^{-/-}$ dKO mice. (**a**) Gating strategy for the identification of Th1 and Th2 cells. Th1 cells were defined as CD3ε^{+} CD4^{+} Tbet^{+}, Th2 cells were defined as CD3ε^{+} CD4^{+} Gata3^{+}; (**b**, **d**, **f**, **h**) Comparison of cell numbers of CD4^{+} T cells (**b**), CD4^{-} T cells (**d**), Th1 cells (**f**) and Th2 cells (**h**) sorted from the single-cell suspensions from mouse ear skin preparations of *wt*, *Trpa1*$^{-/-}$, *Trpv1*$^{-/-}$ and *Trpa1*$^{-/-}$/*Trpv1*$^{-/-}$ dKO mice; (**c**, **e)** Comparison of the percentage of CD4^{+} T cells (**c**) and CD4^{-} T cells (**e**) in the CD45^{+} population from the ear preparations of *wt*, *Trpa1*$^{-/-}$, *Trpv1*$^{-/-}$ and *Trpa1*$^{-/-}$/*Trpv1*$^{-/-}$ dKO mice; (**g**, **i**) Comparison of the percentages of Th1 cells (**g**) and Th2 cells (**i**) in the CD4^{+} population from the ear preparations of *wt*, *Trpa1*$^{-/-}$, *Trpv1*$^{-/-}$ and *Trpa1*$^{-/-}$/*Trpv1*$^{-/-}$ dKO mice. All data are presented as mean ± SEM. n=3 for each group. n.s, not significant, ANOVA.

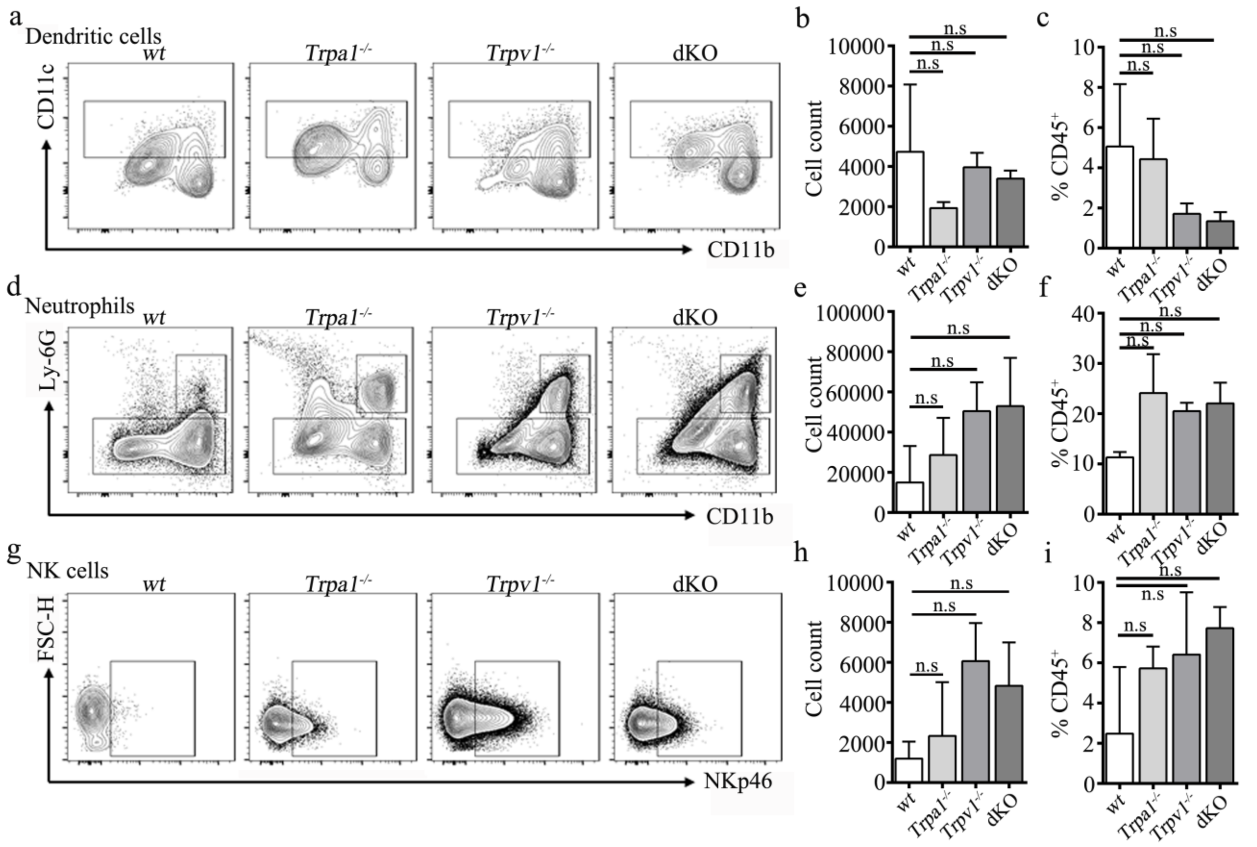

Supplementary Figure 9. Quantification of different types of myeloid cells in single-cell suspensions from SADBE-treated ear skin preparations of *wt, Trpa1*$^{-/-}$*, Trpv1*$^{-/-}$ and *Trpa1*$^{-/-}$/*Trpv1*$^{-/-}$ dKO mice. (**a**, **d**, **g**) Representative FACS plots of dendritic cells (**a**), neutrophils (**d**) and NK cells (**g**). Dendritic cells were defined as I-A$^{b\text{-}hi}$ F4/80$^{+/-}$ CD11b$^{+/-}$ CD11c^{+}, neutrophils were defined as CD11b^{+} Ly6-G^{+} I-A^{b-} F4/80^{-}, NK cells were defined as CD3ε^{-} CD4^{-} NKp46^{+}; (**b**, **e**, **h**) Comparison of cell number of dendritic cells (**b**), neutrophils (**e**) and NK cells (**h**) sorted from the ear preparations of *wt*, *Trpa1*$^{-/-}$, *Trpv1*$^{-/-}$ and *Trpa1*$^{-/-}$/*Trpv1*$^{-/-}$ dKO mice. All data are presented as mean ± SEM. n=3 for each group, n.s, not significant, ANOVA; (**c**, **f**, **i**) Comparison of the percentage of dendritic cells (**c**), neutrophils (**f**) and NK cells (**i**) in the CD45^{+} population from the inflamed ear preparations of *wt*, *Trpa1*$^{-/-}$, *Trpv1*$^{-/-}$ and *Trpa1*$^{-/-}$/*Trpv1*$^{-/-}$ dKO mice. All data are presented as mean ± SEM. n=3 for each group. n.s, not significant, ANOVA.

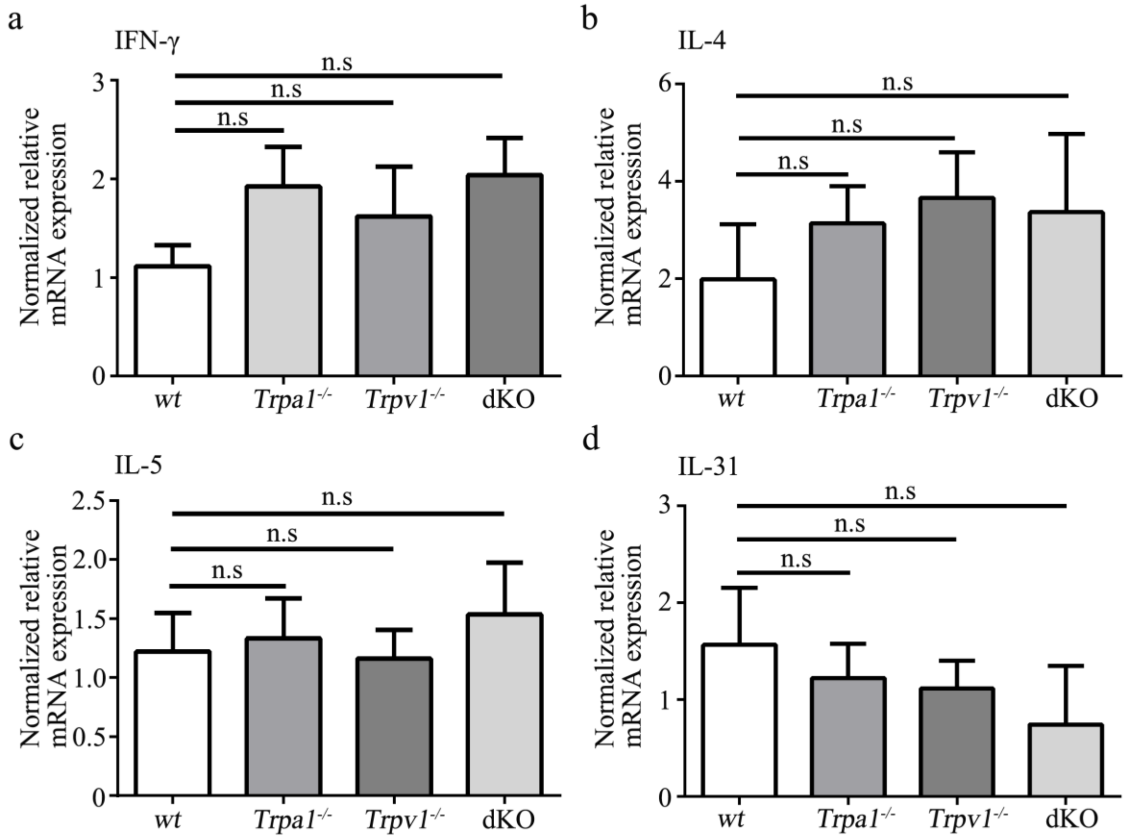

Supplementary Figure 10. Expression levels of Th1 and Th2 cytokines following SADBE treatments are not affected by genetic ablation of TRPA1 and/or TRPV1. (**a-d**) Expression of proinflammatory cytokines IFN-γ (**a**), IL-4 (**b**), IL-5 (**c**), and IL-31 (**d**) in the SADBE-treated ear preparations of *wt*, *Trpa1*$^{-/-}$, *Trpv1*$^{-/-}$ and *Trpa1*$^{-/-}$/*Trpv1*$^{-/-}$ dKO mice. All data are presented as mean ± SEM. n=3 for each group. n.s, not significant, ANOVA.

Supplementary Table 1

EC_{50} values of SADBE-activated responses in wild-type and TRPA1 mutants.

	EC_{50} (mM)	n
TRPA1	1.30±0.02	5
TRPA1-K710A	6.03±0.01*	5
TRPA1-3C	N.D	5
TRPA1-3C+K710A	N.D	5

N.D, not determined; * $p < 0.05$, *versus wt*, Student's *t*-test.

Supplementary Table 2

EC_{50} values of SADBE-activated responses in wild-type and TRPV1 mutants.

	EC_{50} (mM)	n
TRPV1	7.26±0.01	5
TRPV1-R115A	13.79±0.03$^{n.s}$	5
TRPV1-Y512A	N.D	5
TRPV1-S513A	N.D	5
TRPV1-M548L	2.41±0.01**	5
TRPV1-T551A	N.D	5

N.D, not determined; n.s, not significant; ** $p < 0.01$, *versus wt*, Student's *t*-test.
